# Peritumoral ADC values in breast cancer: region of interest selection, associations with hyaluronan intensity, and prognostic significance

**DOI:** 10.1007/s00330-019-06361-y

**Published:** 2019-07-29

**Authors:** Tiia Kettunen, Hidemi Okuma, Päivi Auvinen, Mazen Sudah, Satu Tiainen, Anna Sutela, Amro Masarwah, Markku Tammi, Raija Tammi, Sanna Oikari, Ritva Vanninen

**Affiliations:** 1 Institute of Clinical Medicine, School of Medicine, Oncology, University of Eastern Finland, P.O. Box 1627, FI 70211 Kuopio, Finland; 2 Department of Oncology, Cancer Center, Kuopio University Hospital, P.O. Box 100, FI 70029 Kuopio, Finland; 3 Institute of Clinical Medicine, School of Medicine, Clinical Radiology, University of Eastern Finland, P.O. Box 1627, FI 70211 Kuopio, Finland; 4 Department of Clinical Radiology, Diagnostic Imaging Center, Kuopio University Hospital, P.O. Box 100, FI 70029 Kuopio, Finland; 5 Institute of Biomedicine, University of Eastern Finland, P.O. Box 1627, FI 70211 Kuopio, Finland

**Keywords:** Breast cancer, Diffusion-weighted MRI, Hyaluronan, Lymphatic metastasis, Prognostic factors

## Abstract

**Objectives:**

We aimed to evaluate the differences in peritumoral apparent diffusion coefficient (ADC) values by four different ROI selection methods and to validate the optimal method. Furthermore, we aimed to evaluate if the peritumor-tumor ADC ratios are correlated with axillary lymph node positivity and hyaluronan accumulation.

**Methods:**

Altogether, 22 breast cancer patients underwent 3.0-T breast MRI, histopathological evaluation, and hyaluronan assay. Paired *t* and Friedman tests were used to compare minimum, mean, and maximum values of tumoral and peritumoral ADC by four methods: (M1) band ROI, (M2) whole tumor surrounding ROI, (M3) clockwise multiple ROI, and (M4) visual assessment of ROI selection. Subsequently, peritumor/tumor ADC ratios were compared with hyaluronan levels and axillary lymph node status by the Mann-Whitney *U* test.

**Results:**

No statistically significant differences were found among the four ROI selection methods regarding minimum, mean, or maximum values of tumoral and peritumoral ADC. Visual assessment ROI measurements represented the less time-consuming evaluation method for the peritumoral area, and with sufficient accuracy. Peritumor/tumor ADC ratios obtained by all methods except the clockwise ROI (M3) showed a positive correlation with hyaluronan content (M1, *p* = 0.004; M2, *p* = 0.012; M3, *p* = 0.20; M4, *p* = 0.025) and lymph node metastasis (M1, *p* = 0.001; M2, *p* = 0.007; M3, *p* = 0.22; M4, *p* = 0.015), which are established factors for unfavorable prognosis.

**Conclusions:**

Our results suggest that the peritumor/tumor ADC ratio could be a readily applicable imaging index associated with axillary lymph node metastasis and extensive hyaluronan accumulation. It could be related to the biological aggressiveness of breast cancer and therefore might serve as an additional prognostic factor.

**Key Points:**

*• Out of four different ROI selection methods for peritumoral ADC evaluation, measurements based on visual assessment provided sufficient accuracy and were the less time-consuming method.*

*• The peritumor/tumor ADC ratio can provide an easily applicable supplementary imaging index for breast cancer assessment.*

*• A higher peritumor/tumor ADC ratio was associated with axillary lymph node metastasis and extensive hyaluronan accumulation and might serve as an additional prognostic factor.*

## Introduction

Breast cancer is the most frequently diagnosed cancer and the leading cause of cancer-related deaths in women all around the world [1]. Intense efforts are made to ensure its early detection. Breast cancers exhibit genomic and phenotypic heterogeneity, which is prognostic and influences responses to therapy [2, 3]. In addition, the role of the tumor environment is important because it is the interaction between tumor cells and the surrounding microenvironment which influences tumor evolution and progression [4]. Invasive breast cancers cause increased lymphedema and extracellular matrix remodeling in the area surrounding the tumor [5], and peritumoral edema is one of the recognized features of malignancy.

The peritumoral area comprising the extracellular matrix and various cell types maintains the wound response–like process and inflammation, as well as the increased vascular density and permeability. This represents a distinct microenvironment not only pivotal for tumor progression but also with a significant prognostic potential [6, 7]. Hyaluronan (HA) is an important glycosaminoglycan present in the pericellular and extracellular matrix; HA exists in most normal tissues and participates in many cellular processes like proliferation, migration, and inflammation [8]. HA has the ability to bind large quantities of water molecules, and this hydrophilic feature is an important component of its function [9]. The level of HA is markedly increased in many carcinomas [10], and this can promote tumor progression in several ways [11]. In breast cancer, a high concentration of HA in the pericellular stroma and carcinoma cells strongly associates with poor differentiation of tumors, axillary lymph node positivity, and an unfavorable outcome of the disease [12, 13].

Breast MRI is nowadays an integral part of the diagnostic work-up of tumors. The addition of diffusion-weighted imaging (DWI) in conjunction with routine breast MRI sequences has been shown to improve the specificity of the diagnosis and to assist in lesion characterization [14, 15]. DWI exploits the random motion of water molecules, which can be used to assess the extent of intratumoral tissue cellularity and to detect the presence of an intact cell membrane [16]. The impedance of diffusion of water molecules can be quantitatively estimated by the apparent diffusion coefficient (ADC) value, which provides a more accurate estimation of the cellularity of the tumor microenvironment by minimizing the vascular contribution [17].

Although several studies have examined the associations between peritumoral ADC values and the biological and histological features of breast cancers, region of interest (ROI) selection methods for ADC measurements in the peritumoral area have not been standardized. As a result, a range of different ROI selection methods have been used to measure peritumoral ADC values [18–21]. Although Mori et al [20] showed that the peritumor/tumor ADC ratios could be more diagnostic than peritumoral ADC values themselves when evaluating the lymphovascular invasion status, as far as we are aware, no predictive model exists that connects peritumor/tumor ADC ratios with axillary lymph node status.

HA is known to have a high water-retaining capacity, and DWI in breast MRI measures the random motion of water molecules. Hence, it could be assumed that HA is involved in the peritumoral edema of biologically aggressive cancers and that there is a positive correlation between increased HA levels and peritumoral ADC values due to increased water content.

The purpose of this study was to evaluate the differences in peritumoral ADC values obtained by four different ROI selection methods and to determine the optimal method. Another aim was to evaluate if the peritumor/tumor ADC ratios associate with axillary lymph node positivity and HA accumulation in the peritumoral stroma and breast carcinoma cells.

## Materials and methods

This study included 22 women treated in our tertiary hospital (catchment area of 260,000 inhabitants) between the years 2013 and 2015. All patients were radiologically and histologically diagnosed with invasive breast cancer, and the inclusion criteria for the study were a minimum tumor size of 10 mm on mammography and/or ultrasound. All the patients underwent bilateral 3.0-T breast MRI, and at the time of diagnostic biopsy, three extra snap-frozen core needle biopsies were obtained as published before [22]. Informed consent was obtained from all patients prior to any procedures. The study was approved by the Kuopio University Hospital Research Ethics Board, and all clinical investigations have been conducted according to the relevant guidelines and the principles expressed in the Declaration of Helsinki.

### Samples and hyaluronan measurements

All tumor samples were obtained during the preoperative ultrasound-guided core needle biopsy (G14) 2–4 weeks before the surgery. An automated core needle gun with a 22-mm throw (Bars Magnum, Bard Biopsy Systems) was routinely placed beside the tumor, with at least a minimum of 2–3-mm distance away from the edge of the tumor in order to always include the transitional area between the normal breast tissue and the tumor margin. Any cystic or possible necrotic areas of the tumor were avoided. The samples were immediately snap-frozen in liquid nitrogen. The biopsies were not microdissected for individual cells and hence contained both pericellular stroma and breast carcinoma cells. The HA content was evaluated by an ELISA-like method, which has been described in detail previously [22, 23]. The hyaluronan content was normalized to tissue weight and dichotomized into two groups according to the median of 3 ng/mg.

### MRI acquisition

MRI examinations were performed in the prone position with a 7-element phased-array coil dedicated to breast imaging (Philips Achieva 3.0-T TX, Philips N.V.). The structural breast MRI protocol consisted of five sequences: (1) T1-weighted fast field echo (TR = 4.58 ms; TE (in phase) = 2.3 ms; in-plane resolution 0.48 mm × 0.48 mm; 257 slices; slice thickness 0.7 mm; scanning time 6 min 11 s); (2) T2-weighted turbo spin echo (TR = 5000 ms; TE = 120 ms, flip angle 90°; in-plane resolution 0.6 mm × 0.6 mm; 85 slices; slice thickness 2 mm; scanning time 3 min 20 s); (3) short T1-inversion recovery/turbo spin echo (TR = 5000 ms; TE = 60 ms; TI 230 ms; in-plane resolution 1 mm × 1 mm; 90 slices; slice thickness 2 mm; scanning time 5 min 40 s); (4) a dynamic eTHRIVE sequence (TR = 4.66 ms; TE = 2.3 ms; spectrally adiabatic inversion recovery (SPAIR) fat suppression; dynamic scan time 58.5 s; in-plane resolution 0.96 mm × 0.96 mm; 180 slices; slice thickness 1 mm; with precontrast and six phases after the gadoterate meglumine (0.2 ml/kg, 3 ml/s) injection followed by a saline chaser); and (5) DWI echo planar imaging (TR = shortest; TE = 95 ms; flip angle 90°; SPAIR fat suppression; in-plane resolution 1.15 mm × 1.15 mm; 30 slices; slice thickness 4 mm; diffusion gradients in three directions; scanning time 4 min 8 s) with five respective *b* factors (0, 200, 400, 600, and 800 s/mm^2^). The ADC maps were automatically calculated linearly by the method provided by the MRI vendor.

### Measurement of ADC values

T1-weighted, T2-weighted, and dynamic contrast–enhanced images were referred, and a crosshair tool (Sectra PACS, version 15.1.20.2, Sectra Workstation IDS7) was used to locate the lesion and to correctly position the intratumoral ROI on ADC maps. The intratumoral ROIs were placed on ADC maps with a definitive demarcation from the parenchyma and fat. The ROI was drawn polygonally to cover the entire lesion on the slice with the largest tumor diameter while cystic, necrotic, fatty, and hemorrhagic areas were carefully avoided (ROItumor).

**Fig. 1:**
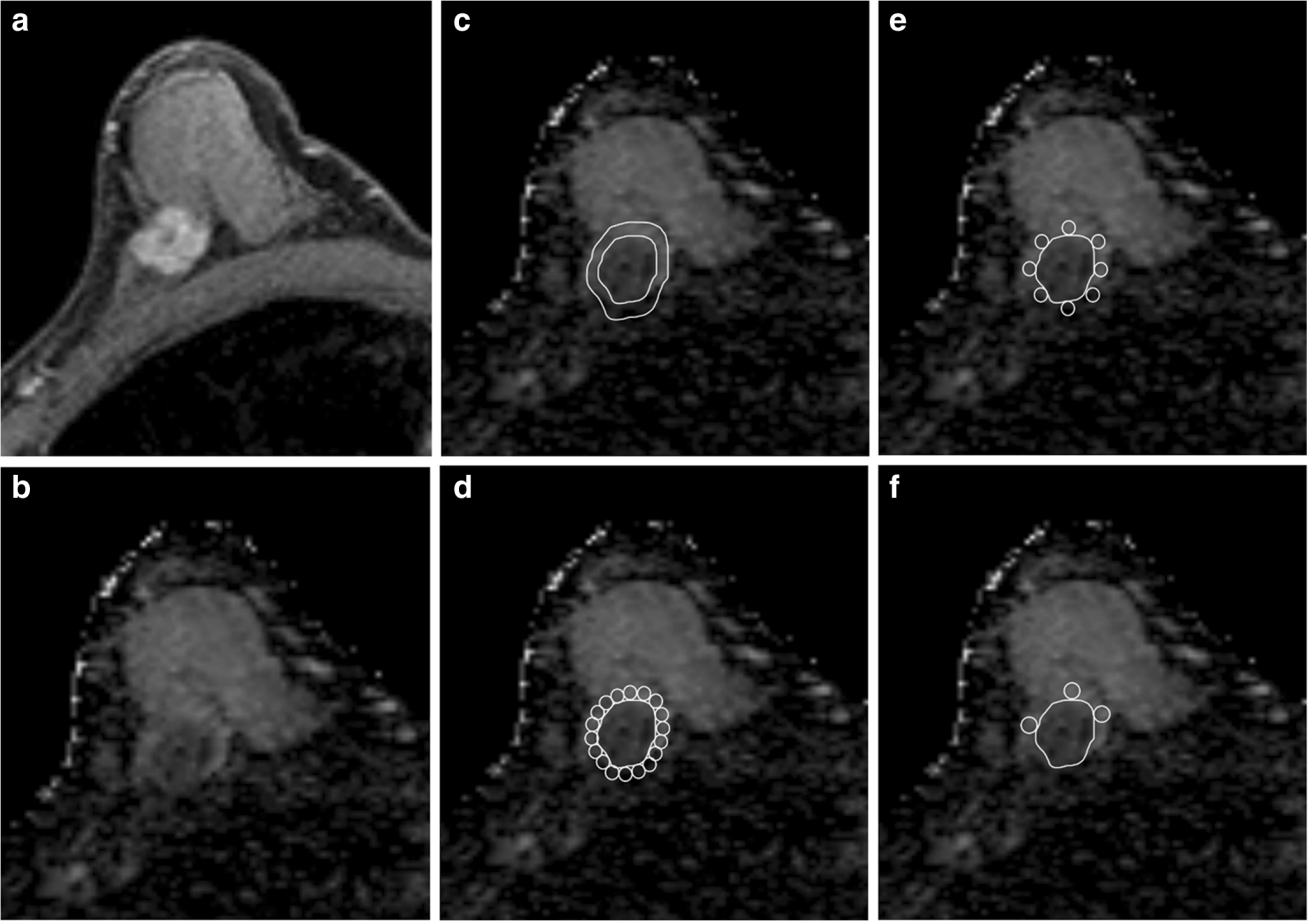
Methods to measure peritumoral ADC values in a regular-shaped oval tumor. **a** T1-weighted gadolinium-enhanced thin-slice source image showing the oval mass lesion with parenchymal background. **b** Apparent diffusion coefficient (ADC) map corresponding to the mass lesion. **c** Method 1 (M1): band ROI method, a band ROI generated adjacent to the tumor border on ADC to cover the whole tumor’s surrounding area. **d** Method 2 (M2): whole tumor surrounding ROI method, circular ROIs placed next to each other adjacent to the tumor border to cover the whole tumor’s surrounding area. **e** Method 3 (M3): clockwise ROI method, 8 circular ROIs placed clockwise at the 0:00, 1:30, 3:00, 4:30, 6:00, 7:30, 9:00, and 10:30 o’clock positions. **f** Method 4 (M4): visual assessment ROI method, 3 circular ROIs placed according to the visually perceived most increased ADC areas

**Fig. 2:**
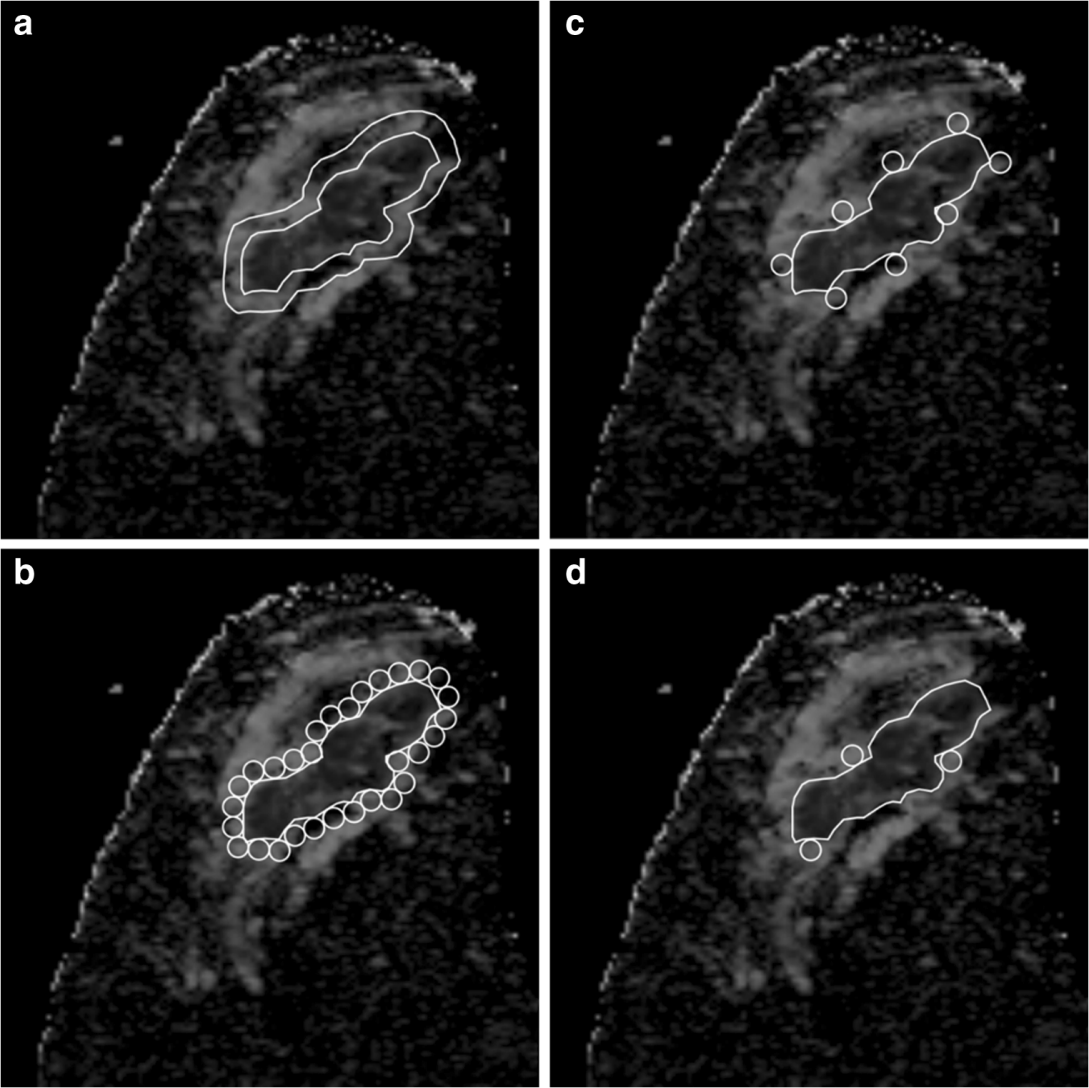
Methods to measure peritumoral ADC values in an irregular-shaped tumor. The descriptions of methods (M1–M4) are identical as in Fig. 1. Irregular tumors are challenging for M3 due to the difficulties to standardize the on-a-clock positions, and are further difficult to reproduce

The peritumoral ADC measurements were performed using four different ROI selection methods: method 1 (M1), a band ROI method first described by McLaughlin et al [19], with a 2-pixel width which was generated by using the ImageJ software (open-source software supported by the NIH) adjacent to the tumor border to cover the area surrounding the tumor; method 2 (M2), the whole tumor surrounding ROI method where 10-pixel-sized round ROIs were placed next to each other adjacent to the tumor border to cover the area surrounding the tumor; method 3 (M3), a simplified modification, the clockwise ROI method where eight round ROIs with 10-pixel size were placed clockwise at 0:00, 1:30, 3:00, 4:30, 6:00, 7:30, 9:00, and 10:30 positions; and method 4 (M4)., a visual assessment ROI method, first described by Mori et al [20], where three round ROIs each of 10-pixel size were placed where the ADC values visually appeared to be most increased on the breast parenchymal tissue adjacent to the tumor border. M1 was measured with ImageJ, while M2, M3, and M4 were measured directly from the picture archiving and communication systems (PACS). A schematic illustration of the four different peritumoral ROI selection methods is shown in Figs. 1c–f and 2a–d.

A breast radiologist (with 10 years of experience in breast MRI analysis) and a breast oncologist (with four years of experience in breast MRI analysis) independently measured the intratumoral and peritumoral ADC values blinded to information about histopathology and hyaluronan metabolism. Minimum, mean, and maximum values of intratumoral ADC were recorded and designated as ADCtmin, ADCtmean, and ADCtmax, respectively.

The four different methods for peritumoral area yielded various amounts of ROIs. In each peritumoral ROI, minimum, maximum, and mean ADC values were first recorded. The lowest minimum and highest maximum values in each method were selected for further analysis and designated as ADCpmin and ADCpmax, respectively. M1 yielded only one peritumoral ROI and the mean values were recorded and designated as ADCpmean. The mean values of M2–4 were averaged and also designated as ADCpmean. M4 intended to identify the maximum ADC values rather than represent the whole peritumoral area, and thus, for statistical comparison, we included only ADCpmax.

For the correlations with HA and lymph node metastasis (LNM), we could not use the highest mean from M2–4 as Mori et al [20] did because M1 gave only the mean value of the whole peritumoral area. Previous results showed that the peritumor/tumor ADC ratios could be more diagnostic than peritumoral ADC values themselves [20]. For the correlation with HA and LNM, we used ADCpmax/ADCtmean ratio.

### Statistical analysis

The statistical analyses were performed using SPSS version 22 (IBM Corporation). The ADC values were evaluated as continuous dependent variables.

Intra- and interobserver reproducibility was evaluated using the interclass correlation coefficients (ICCs). An *r* of 1.0 was considered to indicate perfect agreement; 0.81–0.99, almost perfect agreement; 0.61–0.80, substantial agreement; 0.41–0.60, moderate agreement; 0.21–0.40, fair agreement; and ≤ 0.20, slight agreement [24].

Paired *t* test was performed for the comparison of tumoral ADC values between measurements with PACS and ImageJ. The Friedman test was used for comparison of peritumoral ADC values between the four ROI methods. The Mann-Whitney *U* test was utilized for comparisons of peritumor/tumor ADC ratios with HA status and axillary lymph node status. *p* values less than 0.05 were considered to be statistically significant.

## Results

Twenty-two women (mean age 56.6 ± 11.5 years, range 38–75 years) with 22 invasive breast cancers were analyzed. Mean size of the lesions at histology was 27.0 ± 18.9 mm (range 6.0–90.0 mm). Descriptions of the patient profile and tumor characteristics are presented in Table 1. One patient underwent neoadjuvant therapy before surgery. In this subject, 3.0-T breast MRI and the histopathological analyses (ER/PR, HER2, and Ki-67) were performed before neoadjuvant therapy.

**Table 1:** Patient profile and tumor characteristics

	*N* (%)
Patients	22
Age (years)	56.6 ± 11.5
BMI	25.26 ± 4.54
Tumor stage*	
pT1	10(45.5)
pT2	10 (45.5)
pT3	1 (4.5)
pT4	0 (0)
Axillary node classification*	
pN0	12 (54.5)
pN1	4 (18.2)
pN2	3 (13.6)
pN3	2 (9.1)
Histological grade*	
G1	3 (13.6)
G2	10 (45.5)
G3	8 (36.4)
HER2	
Positive	5 (22.7)
Negative	17 (77.2)
Estrogen receptor	
Positive	21 (95.5)
Negative	1 (4.5)
Progesterone receptor	
Positive	19 (86.4)
Negative	3 (13.6)
Ki-67 expression	
≤ 20%	7 (31.8)
> 20%	15 (68.2)

*BMI* body mass index

*Data from one patient missing due to neoadjuvant chemotherapy

In the evaluation of the agreement within and across readers, it was evident that ICCs for the intratumoral ADC and peritumoral ADC obtained by M2 and M4 exceeded 0.81, indicating almost perfect agreement. The intrarater and interrater ICCs for peritumoral ADC obtained with M3 were only 0.520 and 0.312, respectively, indicating moderate agreement (Tables 2 and 3).

**Table 2:** Intraclass correlation coefficients (ICCs) of ADC

	ICC (95% confidence interval)
Tumoral ADC	0.960 (0.906–0.983)
Peritumoral ADC	
Method 2	0.945 (0.869–0.977)
Method 3	0.520 (−0.139–0.799)
Method 4	0.954 (0.891–0.981)

**Table 3:** Interclass correlation coefficients (ICCs) of ADC by two readers

	ICC (95% confidence interval)
Tumoral ADC	0.951 (0.885–0.979)
Peritumoral ADC	
Method 2	0.978 (0.948–0.991)
Method 3	0.312 (−0.116–0.643)
Method 4	0.957 (0.900–0.982)

Table 4 summarizes the comparison of the four ROI selection methods. No significant difference was found depending on whether minimum, mean, or maximum values of tumoral ADC were measured by ImageJ or local PACS. No significant difference was observed in the maximum values of peritumoral ADC between any of the four methods. No significant difference was found in the minimum or mean values of peritumoral ADC according to M1–3. M4 was the less time-consuming method (Table 5).

**Table 4:** Summary of ADC values (× 10^−3^ mm^2^/s) by four ROI selection methods

	Method 1	Method 2	Method 3	Method 4	*p* value
ADCtmin	0.17 ± 0.16	0.13 ± 0.15	0.13 ± 0.15	0.13 ± 0.15	0.19*
ADCtmean	0.61 ± 0.15	0.63 ± 0.13	0.63 ± 0.13	0.63 ± 0.13	0.20*
ADCtmax	1.19 ± 0.21	1.29 ± 0.26	1.29 ± 0.26	1.29 ± 0.26	0.051*
ADCpmin	0.00 ± 0.01	0.02 ± 0.07	0.02 ± 0.08	0.94 ± 0.32	0.529^¤^
ADCpmean	0.74 ± 0.21	0.70 ± 0.21	0.71 ± 0.21	1.01 ± 0.30	0.066^¤^
ADCpmax	1.53 ± 0.21	1.59 ± 0.36	1.53 ± 0.37	1.56 ± 0.33	0.087^#^

The data are summarized as mean ± SD

*ADC* apparent diffusion coefficient

ADCtmin, ADCtmean, ADCtmax: minimum, mean, and maximum values of tumor ADC, respectively; ADCpmin, ADCpmean, ADCpmax: minimum, mean, and maximum values of peritumor ADC, respectively

*Paired *t* test

^¤^The Friedman test; data from method 4 was not used for correlation

^#^The Friedman test

**Table 5:** Average time required for measurement

Peritumoral ADC	Time in seconds
Method 2	211.5 ± 121.0
Method 3	73.0 ± 11.1
Method 4	35.0 ± 7.4

Table 6 summarizes the associations between peritumor/tumor ADC ratios and HA quantity and axillary lymph node metastasis. Statistically significant associations were found between both HA quantity and lymph node metastasis with the measurements obtained from M1, M2, and M4. In all ROI selection methods, peritumor/tumor ADC ratios tended to be greater when the HA content determined by ELISA-like assay was high and when axillary lymph node metastasis was positive.

**Table 6:** Association between peritumor/tumor ADC ratios and HA intensity and lymph node metastasis

	Method 1		Method 2		Method 3		Method 4	
	ADC ratio	*p* value	ADC ratio	*p* value	ADC ratio	*p* value	ADC ratio	*p* value
HA quantity								
Low	2.15 ± 0.33	0.004	2.21 ± 0.40	0.012	2.36 ± 0.49	ns	2.20 ± 0.42	0.025
High	3.11 ± 1.06		2.87 ± 0.72		2.65 ± 0.76		2.79 ± 0.64	
Lymph node metastasis								
Negative	2.26 ± 0.46	0.001	2.29 ± 0.37	0.007	2.33 ± 0.42	ns	2.30 ± 0.37	0.015
Positive	3.26 ± 1.02		3.05 ± 0.65		2.77 ± 0.78		2.94 ± 0.62	

*ns* not significant

## Discussion

In this study, we compared the minimum, mean, and maximum values of peritumoral ADC values derived from four different ROI selection methods. Our results revealed that neither minimum, mean, nor maximum ADC values were significantly different between the ROI selection methods. We further correlated peritumor/tumor ADC ratios with the amount of HA and axillary lymph node status. Higher peritumor/tumor ADC ratios were associated with a high HA content and positive lymph node metastasis, which are both indicators for a poor prognosis.

### ROI measurement validations

Although peritumoral ADC values have been attracting more attention as a prognostic factor for breast [18–21, 25], hepatic [26], and endometrial cancers [27], the optimal method for ROI selection is not known. The first method used in this study, the band ROI method (M1), has been previously applied in several studies [18, 19, 21, 25]. This method automatically processes almost all of the steps and does not require any specialized technique or hard-to-learn analyzing skills, which ensures straightforward access. The band ROI method also covers the whole peritumoral area in question and instantaneously gives minimum, mean, and maximum values, providing a very quick measurement. However, it does take time and effort to transfer the data from the local PACS to the separate ImageJ software, making it more difficult to apply in daily clinical practice.

In contrast, the visual assessment ROI method (M4) used by Mori et al [20] enables measurements on the local PACS software, requires no special sophisticated software function, and demands that only three ROIs have to be placed, which is less time-consuming and feasible in daily clinical practice. However, we were skeptical about the accuracy of visually selecting the highest ADC values, and suspected that the results might be operator-dependent based on the reader’s proficiency in assessing breast MR images. We therefore undertook the whole tumor surrounding ROI method (M2) in order to comprehensively measure the whole peritumoral area on local PACS, as well as to examine the clockwise 8-ROI method (M3) as its simpler modification, and compared the values obtained from those methods.

First, we confirmed that none of the minimum, mean, or maximum values of tumoral ADC showed significant differences between ImageJ and the local PACS, which indicated that these image processing programs should produce similar results. We then confirmed that the peritumoral ADC values obtained by the clockwise ROI method (M3) were equivalent to those obtained by the ROI methods surrounding the whole tumor (M2). Most importantly, the results show that only three ROIs obtained by the visual assessment ROI method (M4) were sufficient to identify the needed values. Furthermore, only the clockwise ROI method (M3) failed to provide significant results when examining the associations between peritumor/tumor ADC ratios and HA status. This can be explained by the fact that the clockwise ROI method places a limited number of peritumoral ROIs regardless of their intensities, and this caused a failure in identifying those areas with the highest representative ADC values (Figs. 1e and 2c). In contrast, the visual assessment ROI method (M4) succeeded in overcoming this obstacle and obtained the highest ADC values while requiring even fewer ROIs.

There was only a moderate ICC for peritumoral ADC measurements obtained from the clockwise method (M3). This can be explained by the fact that clockwise ROIs were difficult to place when tumor shapes were irregular (Fig. 2c). These moderate values were mostly due to the difficulty of placing ROIs on irregularly shaped tumors, where even marking the starting 12 o’clock position may prove to be a difficult task. However, the visual assessment ROI method (M4) gave almost perfect ICC, which indicates that this method is effective regardless of the reader’s experience in analyzing breast MR images. Based on our results, we conclude that visual assessment ROI measurements represent the less time-consuming and sufficiently accurate evaluation method for identifying the peritumoral area.

### Peritumor/tumor ADC ratios

Although ADC has been widely investigated as an imaging prognostic biomarker, the limited reproducibility of ADC values across different imaging vendors, field strengths, and imaging centers is controversial [28–30]. Therefore, we agree with the previous conclusions that the peritumor/tumor ADC ratio is more reliable and generally more applicable than measuring only the peritumoral area [18], and subsequently reduces bias. Furthermore, while peritumoral high intensity signals visualized on T2-weighted images were consistently shown to be associated with biologically more aggressive diseases [31, 32], this sign has only been visualized in a small portion of tumors (15–32%) [31–33]. Accordingly, measuring peritumor/tumor ADC ratio can increase the diagnostic accuracy and efficiently evaluate the peritumoral area even in the absence of any visually obvious edema.

### HA and LNM associations

It was previously established that HA levels increase in most forms of inflammation, including those associated with the progression of malignant tumors. HA is a known promoter of breast cancer and other malignancies and its abundance is an indicator of an aggressive tumor type and cancer progression [12, 13]. Since HA is increased in edema and contributes to tissue hydration, we hypothesized that the HA content might be associated with the ADC ratio values. Indeed, breast cancers with a higher HA content did reveal significantly greater peritumor/tumor ADC ratios. The positive correlations of peritumor/tumor ADC ratio with lymph node positivity and HA content suggest that the ADC ratio may prove to be a valuable, non-invasive prognostic indicator available even in the preoperative setting. However, this will need to be verified in a larger patient population and with a sufficient period of follow-up.

### Limitations

We wanted to correlate the ADC values with tumor HA content. The assay of the HA is quite unique and relatively laborious; this meant that only a limited number of patients could be included in our analysis. Nevertheless, statistically significant results were successfully obtained; i.e., this is the first time that the association between breast tumor HA content and peritumor/tumor ADC ratios has been determined.

To conclude, the validation of the four ROI selection methods showed that all of them indicated relatively well the peritumoral ADC values of breast cancers, but a reliable result could be obtained by selecting three ROIs according to visual evaluation by the reader. The findings suggest that peritumor/tumor ADC ratios could feasibly be an applicable imaging index of the aggressiveness and prognosis of breast cancers.

## Abbreviations

ADC: Apparent diffusion coefficient
DWI: Diffusion-weighted imaging
HA: Hyaluronan
ICC: Interclass correlation coefficient
LNM: Lymph node metastasis
MRI: Magnetic resonance imaging
PACS: Picture archiving and communication systems
ROI: Region of interest
